# COVID-19 healthcare demand and mortality in Sweden in response to non-pharmaceutical mitigation and suppression scenarios

**DOI:** 10.1093/ije/dyaa121

**Published:** 2020-09-20

**Authors:** Henrik Sjödin, Anders F Johansson, Åke Brännström, Zia Farooq, Hedi Katre Kriit, Annelies Wilder-Smith, Christofer Åström, Johan Thunberg, Mårten Söderquist, Joacim Rocklöv

**Affiliations:** d1 Department of Public Health and Clinical Medicine, Section of Sustainable Health, Umeå University, Umeå, Sweden; d2 Department of Clinical Microbiology and the Laboratory for Molecular Infection Medicine Sweden, Umeå University, Umeå, Sweden; d3 Department of Mathematics and Mathematical Statistics, Umeå University, Umeå, Sweden; d4 Evolution and Ecology Program, International Institute for Applied Systems Analysis, Laxenburg, Austria; d5 Department of Epidemiology and Global Health, Umeå University, Umeå, Sweden; d6 Department of Disease Control, London School of Hygiene and Tropical Medicine, London, UK; d7 Heidelberg Institute of Global Health, University of Heidelberg, Heidelberg, Germany; d8 Department of Surgical and Perioperative Sciences, Anesthesiology and Intensive Care Medicine, Umeå University, Umeå Sweden

**Keywords:** COVID-19, corona virus, SARS-CoV-2, epidemiology, epidemic, outbreak, pandemic, infections, care demand, intensive care demand, deaths, mortality, excess mortality, Sweden

## Abstract

**Background:**

While the COVID-19 outbreak in China now appears suppressed, Europe and the USA have become the epicentres, both reporting many more deaths than China. Responding to the pandemic, Sweden has taken a different approach aiming to mitigate, not suppress, community transmission, by using physical distancing without lockdowns. Here we contrast the consequences of different responses to COVID-19 within Sweden, the resulting demand for care, intensive care, the death tolls and the associated direct healthcare related costs.

**Methods:**

We used an age-stratified health-care demand extended SEIR (susceptible, exposed, infectious, recovered) compartmental model for all municipalities in Sweden, and a radiation model for describing inter-municipality mobility. The model was calibrated against data from municipalities in the Stockholm healthcare region.

**Results:**

Our scenario with moderate to strong physical distancing describes well the observed health demand and deaths in Sweden up to the end of May 2020. In this scenario, the intensive care unit (ICU) demand reaches the pre-pandemic maximum capacity just above 500 beds. In the counterfactual scenario, the ICU demand is estimated to reach ∼20 times higher than the pre-pandemic ICU capacity. The different scenarios show quite different death tolls up to 1 September, ranging from 5000 to 41 000, excluding deaths potentially caused by ICU shortage. Additionally, our statistical analysis of all causes excess mortality indicates that the number of deaths attributable to COVID-19 could be increased by 40% (95% confidence interval: 0.24, 0.57).

**Conclusion:**

The results of this study highlight the impact of different combinations of non-pharmaceutical interventions, especially moderate physical distancing in combination with more effective isolation of infectious individuals, on reducing deaths, health demands and lowering healthcare costs. In less effective mitigation scenarios, the demand on ICU beds would rapidly exceed capacity, showing the tight interconnection between the healthcare demand and physical distancing in the society. These findings have relevance for Swedish policy and response to the COVID-19 pandemic and illustrate the importance of maintaining the level of physical distancing for a longer period beyond the study period to suppress or mitigate the impacts from the pandemic.

**Key Messages**

- We find that physical distancing and isolation of infectious individuals without lockdown is effective in mitigating much of the negative direct health impact from the COVID-19 pandemic in Sweden, but has a higher death toll compared with other Scandinavian countries who did implement a lockdown.
- It appears that Sweden has managed to ensure, by implementation of physical distancing initiated from the end of March, that intensive care unit (ICU) demands do not exceed ICU capacities and that deaths are substantially reduced compared to several alternative scenarios.
- In the counterfactual scenario, the intensive care unit demand is estimated to be ∼20 times higher than the intensive care capacity in Sweden and the number of deaths would be between 40 000 to 70 000.
- Under current mitigation strategies, the health impacts, and their associated cost are, however, still substantial, and are likely to continue to rise unless the virus is suppressed, or eliminated. In the mitigation and suppression scenarios, including the scenario fitting best to data from Sweden by the end of May 2020, there is an obvious risk of resurgence of the epidemic unless physical distancing, shielding of the elderly and home isolation are effectively sustained.
- A statistical analysis of excess mortality for all causes of death indicates that the number of deaths attributed to COVID-19 could be increased by 40%.

## Introduction

The novel SARS-CoV-2 is highly transmissible.^1^ It has rapidly spread around the globe since it first emerged in Wuhan, China,^2^ at a rate much faster than other emerging infectious diseases such as Ebola.^3^ In response to the COVID-19 outbreak, China implemented extraordinary public health measures at great socio-economic cost. They moved swiftly to ensure early identification of cases, with prompt laboratory testing, facility-based isolation of all cases, contact tracing and quarantine.^4^ In the community, physical distancing was implemented at a grand scale, all mobility put to an halt, and the city of Wuhan was in lockdown for about 9 weeks.^5^ China’s tremendous efforts showed success.^6^ Other Asian countries facing a major explosion, such as South Korea, also managed to curb the epidemic. South Korea employed very liberal testing, hospital-based isolation of all cases, combined with extensive contact tracing enhanced by mobile phone and digital technologies, but did not use a lockdown.^7^^,^^8^

While the outbreak in China appears to be contained, since mid-March 2020, the epicentre of the COVID-19 pandemic is in Europe, and since April in the USA. There is thus an urgent need to determine how best to reduce transmission rates, the height of the epidemic peak, the peak demand on healthcare services and how to reduce fatalities.

In the absence of vaccines, a wide range of control measures can be considered to contain or mitigate COVID-19. These include active case finding with prompt isolation of cases, contact tracing with quarantine of contacts, school closures and closures of public places, mobility restrictions, physical distancing in the community, physical distancing only of the elderly and lockdowns (also known as Cordon sanitaire).^4^ There is currently no consensus about which measures should be considered, in which combination, and at which epidemiological threshold such measures should be implemented for maximum public health impact.^9^

Two strategies can be considered: (i) suppression that aims to rapidly reverse epidemic growth, thereby reducing case numbers to low levels, and (ii) mitigation which focuses on slowing but not necessarily immediately stopping epidemic spread—reducing peak healthcare demand while shielding those most at risk of severe disease from infection. Each policy has major challenges. Suppression aims to rapidly reduce the reproduction number, $R_{0}$, to <1, thus causing case numbers to consistently decline. Mitigation aims to slow spread by reducing $R_{0}$ to a value close to but slightly >1 for some time before the epidemic growth will cease gradually, partially in reponse to increasing levels of disease immunity in the population.

Public health measures need to be weighed against economic repercussions and mental illness caused by prolonged lockdown. Although more strict community containment measures such as lockdowns will result in a shorter duration of the outbreak,^10^ the non-health sector negative consequences may be huge.

Sweden decided to implement public health interventions without a lockdown. Schools and universities were not closed, restaurants and bars remained open, instead Swedish citizens implemented ‘work from home’ policies where possible, social distancing without police enforcement and shielding of those >70 years of age.

Here we aim to quantify the effects of the Swedish measures. We estimate the impact of COVID-19 on the Swedish population at the municipality level, considering demography and human mobility under various scenarios of mitigation and suppression. We estimate the time course of infections, health care needs, and mortality in relation to Swedish intensive care unit (ICU) capacity, as well as the costs of care, and compared alternative policies and a counterfactual scenario.

## Methods

We developed a compartmental epidemiological model based on the SEIR (susceptible, exposed, infectious, recovered) formulation, and extended it to account for additional variables including compartments for health and ICU care. All these variables were age-structured (0–59, 60–79 and 80+ years). The model included age-structured compartments for susceptible, exposed, infected, inpatient care, ICU care, dead and recovered populations based on Swedish population data at the municipality level (see Supplementary Information 1, available as Supplementary data at *IJE* online). Overall, the population of infected individuals was divided into two different groups, those that had sufficiently severe symptoms to potentially end up in hospital care (21.6%), and those who had mild or asymptomatic infections or were sick at home (78.4%).^11^^,^^12^ This parameter was calibrated to data. The model allowed for three different ways that deaths could occur: (i) after unsuccessful ICU treatment; (ii) after routine triage and denial of ICU due to low chances of surviving or when ICU demand exceeds ICU capacity; and (iii) outside of healthcare.

The model captured spatial demographic heterogeneities at the level of municipality in Sweden, and inter-municipality travelling based on a radiation model (see Supplementary Information 1). The radiation model was calibrated using a N1H1 Influenza A model and data for the period 2015–18 in Sweden. Demographic data were obtained at the municipality level for the year 2018 from Statistics Sweden.

The parameterization of the model was achieved in two ways: (i) some parameters were set to fixed values based on what is known through literature and data; (ii) some parameters were given initial values from the international literature and then calibrated by fitting the model to current available outbreak-data on infection prevalence, seroprevalence, deaths, ICU load and healthcare in the Stockholm region. The full set of parameter values are given in the Supplementary Table S1.1, available as Supplementary data at *IJE* online. Age-specific health-care need parameters from Ferguson *et al*.^13^ were initially used to represent the three age groups in our study (Supplementary Table S1.2, available as Supplementary data at *IJE* online). These values were then calibrated to better reflect observed infection prevalence, seroprevalence, in-patient care demand, ICU care demand and registered deaths due to COVID-19 from the Stockholm region and Sweden (See Supplementary Table S1.2).

The infectious period is likely to vary by individual and range from days to weeks. Viral shedding is reported to occur from 7 to 22 days, including in mild cases of disease,^14^ and is a driver of disease transmission. Isolation of patients, or staying home if presenting with symptoms, will reduce transmission to contacts and is a key strategy to contain COVID-19. It shortens the period that infected persons are able to infect others. Importantly, transmission from an infected but asymptomatic or pre-symptomatic individual can still occur despite control measures.^15^ We assumed that the average effective infectious period in the general population is 5 days, shortened from 7 days by natural isolation of symptomatic infected individuals. We assumed that individuals going into healthcare were admitted on average after 3 days of symptomatic infection and were isolated from transmitting the virus to other individuals while in hospital care.

We used an observed sample of virus prevalence positivity from the Stockholm region to validate the model predictions. The virus prevalence was measured by nucleic acid detection by a PCR method in nasopharyngeal specimens from the general population in the Stockholm region. Sampling was conducted for about 1 week centred around the 1 April and was managed by the Swedish Public Health Agency. The measurement indicated 2.5% [95% confidence interval (CI) = 1.4–4.2 %] of the population was infected.^16^ To calibrate the infection prevalence in the model with the study result, considering virus is detectable for an average of 12 days, which goes beyond the average infectious period, we adjusted the model by allowing a latent compartment for the period virus is detectable after the infectious period.^17^ We further calibrated the model to samples of seroprevalence estimated in the Stockholm region, estimating 7.3% of the population had cumulatively been exposed to the virus up to the first half of April (7 April).^18^

We calibrated the daily transmission rate, β, to 0.892 c(t) based on Swedish data for the Stockholm region on healthcare load, virus prevalence, seroprevalence and mortality (Supplementary Information 1), where $c\left(t\right)$ is a time dependent contact-rate scaling parameter (Supplementary Information 1). In our model, $R_{0}$ is dependent on contact-rate and infectious period, whose parameters in turn are dependent on age-distribution (see Supplementary Information 1). Accordingly, we account for spatial and demographical heterogeneity in $R_{0}$. Country-level R_0_ values are estimated as an average of the municipality specific R_0_ values. It is not within the scope of the paper to derive exact values for $R_{0}$, yet we can see that the within-municipality $R_{0}$, for the no-countermeasures scenario [i.e. scenario (a), see below], is within the range 2.67–4.45, and this range is consistent with the reported basic reproduction rates for COVID-19.^1^ The country-level $R_{0}$ is given by an average over municipality-local $R_{0}$ values. Due to the many travellers infected with SARS-CoV-2 arriving from Italy, Austria and other parts of Europe, in the week of the 24 February, the model was seeded with 1 case per 100 000 individuals for all municipalities except for the municipalities within the Stockholm region that were seeded with 1 case per 50 000 individuals.

The model was set up to predict the municipality transmission dynamics and inter-municipality spread across Sweden starting from 24 February and ending a little more than 6 months later, 1 September 2020. The model used scenarios to describe the countermeasures and counterfactual impacts. The mitigation and suppression scenario had onset on 20 March by a transient function with full effect by early to mid-April (Supplementary Table S1.1). The five different scenarios are summarized by:

(a) no public health interventions (counterfactual scenario);
(b) modest physical distancing in ages 0–59 years, moderate in ages 60+ years;
(c) modest physical distancing in ages 0–59 years, moderately strong in ages 60+ years;
(d) moderate physical distancing in ages 0–59 years, very strong in ages 60–79 years, strong in ages 80+ years, and with an increased degree of isolation of infectious individuals;
(e) moderate physical distancing in ages 0–59 years, strong in ages 60+ years, and further improved isolation of infectious individuals.

A complete description of the scenarios is provided in the Supplementary Information 1.

In the scenarios the ICU capacity was compared against twice the baseline availability of 526 ICU beds in Sweden, as this was doubled in response to the COVID-19 pandemic. The numbers of deaths and the infection fatality rate (IFR) associated with the different mitigation and supression scenarios were derived from our model. We also derived the direct healthcare cost for each of the scenarios (see Supplementary Information 4, available as Supplementary data at *IJE* online).

Additionally, we extracted total all-cause of deaths from the Stockholm region and made statistical estimates of the excess mortality beyond confirmed COVID-19 cases. This was done by comparing the excess mortality during the COVID-19 outbreak with the mortality the weeks before the outbreak for the same and previous years by applying time series regression methods while adjusting for time trends and the patterns of deaths in previous months and years (Supplementary Information 3, available as Supplementary data at *IJE* online).

## Results

**Figure 1:**
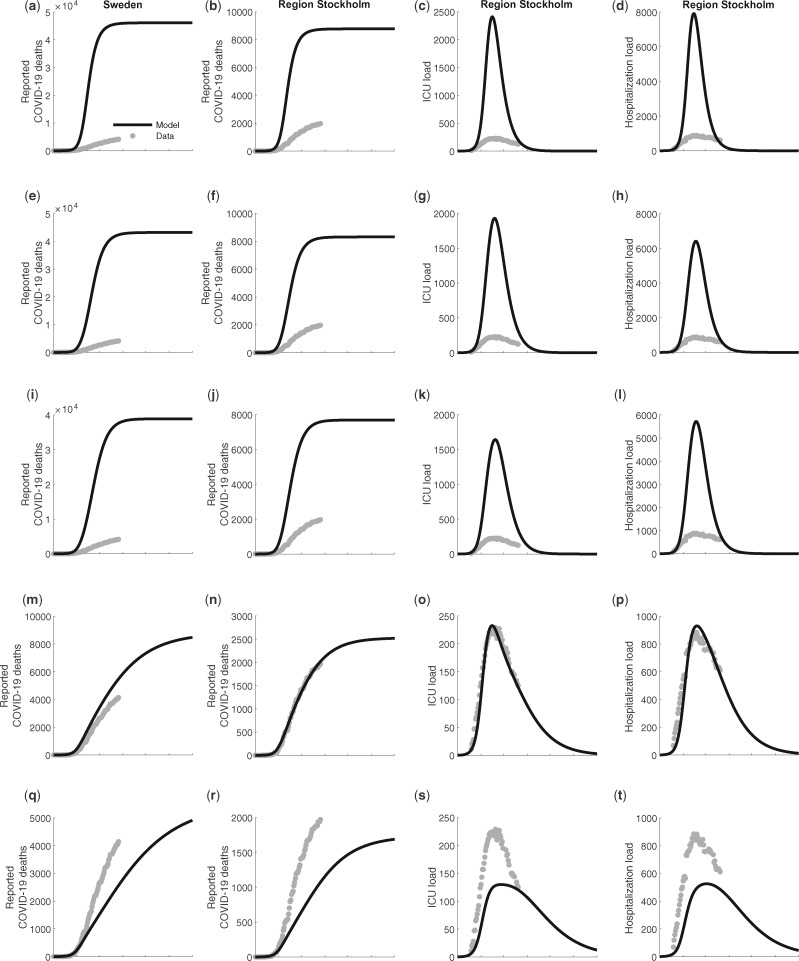
Predicted number of total deaths from COVID-19 in the whole population in Sweden (first column), and for Stockholm region (second column); predicted demand of ICU beds in Stockholm (third column), and; inpatient care beds in Stockholm (fourth column). Actual observations in the early phase of the outbreak are illustrated as circles (O). The scenarios are organized in rows with panel (**a**) no public health interventions (counterfactual scenario); (**b**) modest physical distancing in ages 0–59 years, moderate in ages 60+ years; (**c**) modest physical distancing in ages 0–59 years, moderately strong in ages 60+ years; (**d**) moderate physical distancing in ages 0–59 years, very strong in ages 60–79 years, strong in ages 80+ years, and with an increased degree of isolation of infectious individuals; (**e**) moderate physical distancing in ages 0–59 years, strong in ages 60+ years, and further improved isolation of infectious individuals. Mitigation giving rise to these predicted values had onset the 20th of March.

**Figure 2:**
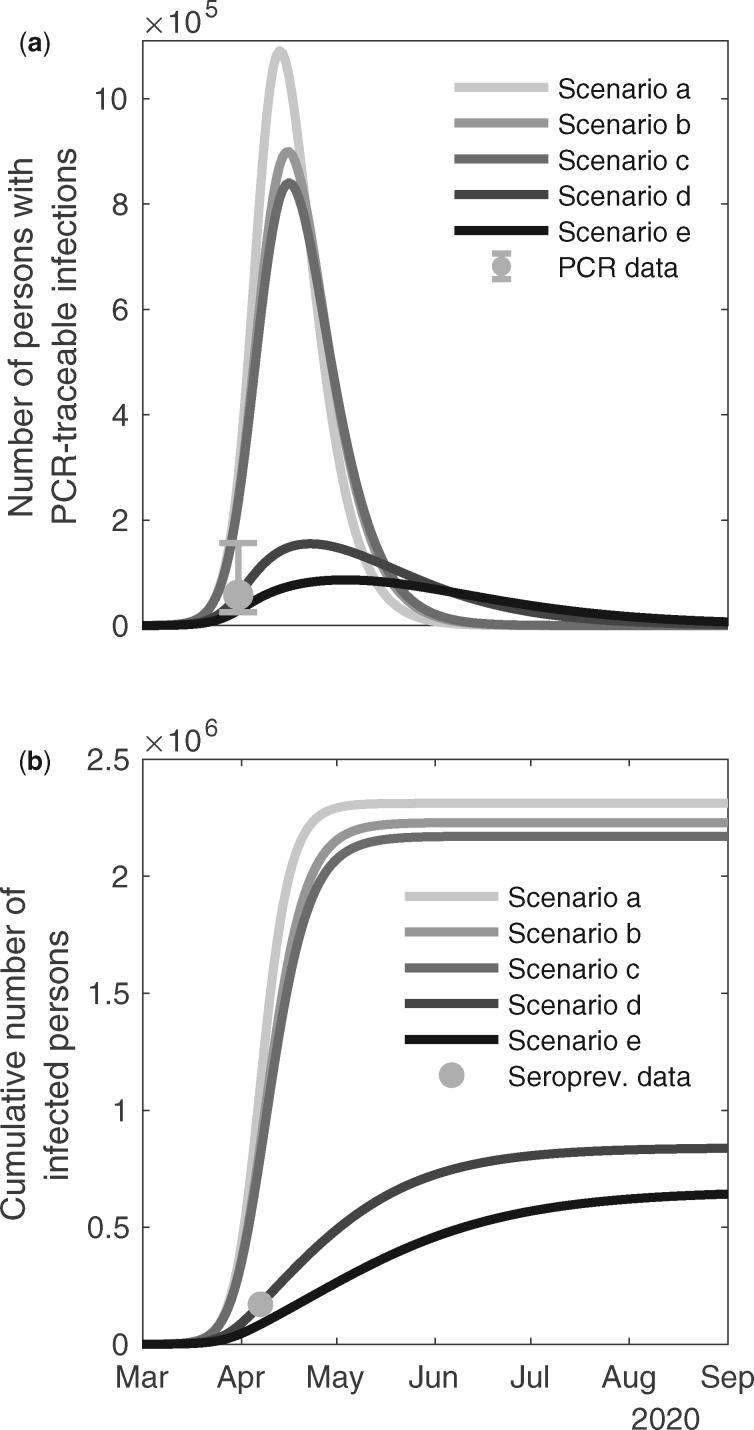
Top panel (**a**) The number of individuals in the Stockholm region predicted to carry the virus over time as determined by a virus detection assay. The empirical measurement from April 1st, 2020, using a population sample, is illustrated by a circle (O) with 95% CI (vertical bars); Bottom Panel (**b**) The cumulative number of predicted infected people detected in an antibody test by the end of April (assuming antibodies cannot be not detected immediately. The scenarios are organized in rows with panel (**a**) no public health interventions (counterfactual scenario); (**b**) modest physical distancing in ages 0–59 years, moderate in ages 60+ years; (**c**) modest physical distancing in ages 0–59 years, moderately strong in ages 60+ years; (**d**) moderate physical distancing in ages 0–59 years, very strong in ages 60–79 years, strong in ages 80+ years, and with an increased degree of isolation of infectious individuals; (**e**) moderate physical distancing in ages 0–59 years, strong in ages 60+ years, and further improved isolation of infectious individuals. Mitigation giving rise to these predicted values had onset the 20th of March.

The model showed good agreement with the reported COVID-19 related deaths in Stockholm up to the end of May 2020, in scenario (d) (Figure 1). In Table 1 we present the *R*^2^ and the mean square error of the observations to the predictions for scenarios (a)–(e) in relation to: deaths for Sweden as a whole, deaths in Stockholm, ICU bed demand in Stockholm and in-patient care in Stockholm. Scenario (d) further describes well the observation that 2.5% of the population in Stockholm was infected with the SARS-CoV-2 virus centred around 1 April, as well as the antibody prevalence (Figure 2). Overall, the IFR for Sweden in scenarios (a)–(e) is estimated to 0.46; 0.44; 0.42; 0.34 and 0.30 %, respectively (Table 1). In Stockholm the IFR is estimated to 0.38, 0.37, 0.35, 0.30, 0.27 % for scenarios (a)–(e) respectively.

**Table 1:** Estimates of *R*^2^ and root-mean-square error (RMSE) for the different model scenarios of observations from Sweden and Stockholm, along with the resulting infection fatality ratio (IFR) for Sweden

Scenario	Deaths Sweden		Deaths Stockholm		ICU Stockholm		In-patient care Stockholm		IFR Sweden (%)
	*R* ^2^	RMSE	*R* ^2^	RMSE	*R* ^2^	RMSE	*R* ^2^	RMSE	
(a)	0.32%	25453.3	2.57%	4484.9	0.28%	1188.3	0.35%	3727.3	0.46
(b)	0.47%	21154.7	3.40%	3877.4	0.40%	994.5	0.48%	3144.7	0.45
(c)	0.66%	17755.8	4.36%	3407.3	0.54%	855.5	0.60%	2818.4	0.42
(d)	83.5%	643.4	99.76%	35.8	94.97%	14.5	83.5%	97.7	0.35
(e)	76.3%	805.9	66.09%	521.3	43.28%	72.1	35.0%	299.3	0.30

**Figure 3:**
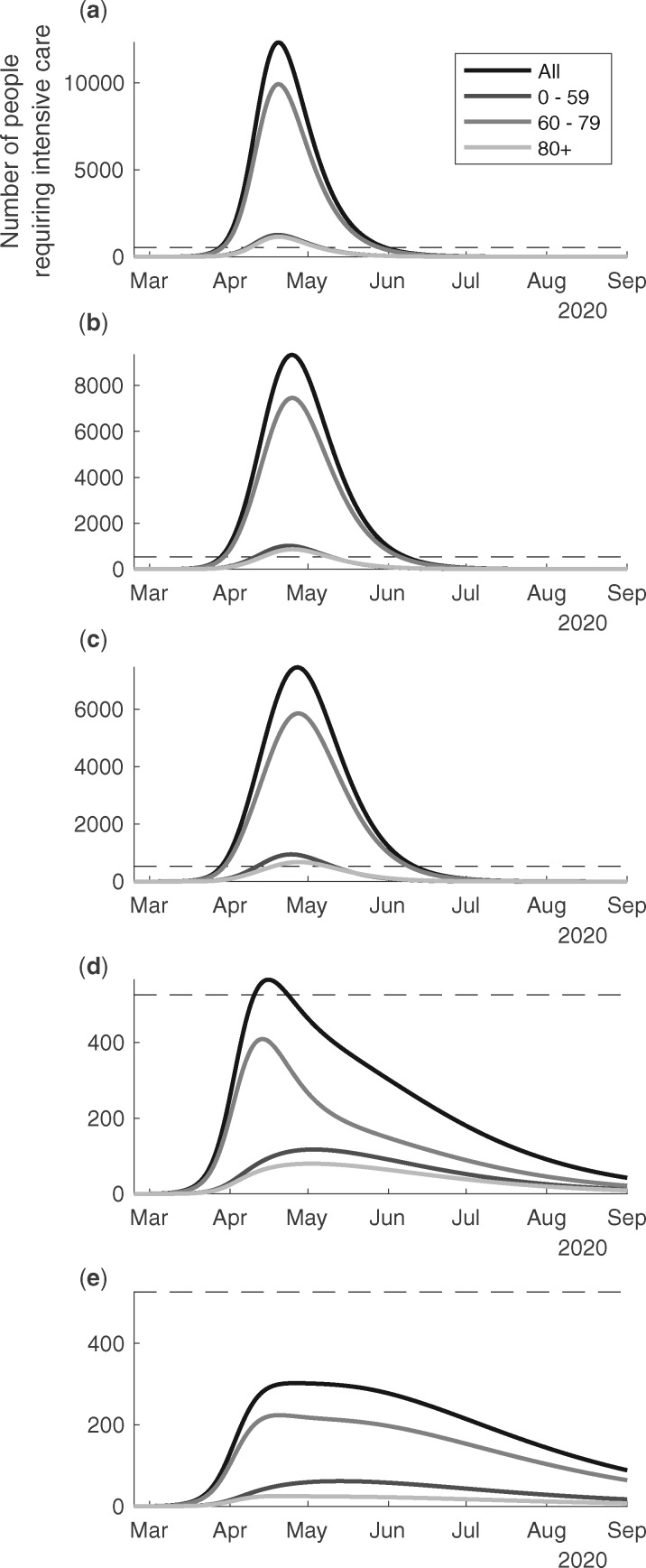
The predicted ICU bed demand per day from 24th of February to the 1st of September, 2020, overall in Sweden in relation to different suppression & mitigation scenarios. The scenarios are organized in rows with panel (**a**) no public health interventions (counterfactual scenario); (**b**) modest physical distancing in ages 0–59 years, moderate in ages 60+ years; (**c**) modest physical distancing in ages 0–59 years, moderately strong in ages 60+ years; (**d**) moderate physical distancing in ages 0–59 years, very strong in ages 60–79 years, strong in ages 80+ years, and with an increased degree of isolation of infectious individuals; (**e**) moderate physical distancing in ages 0–59 years, strong in ages 60+ years, and further improved isolation of infectious individuals. Mitigation giving rise to these predicted values had onset the 20th of March.

**Figure 4:**
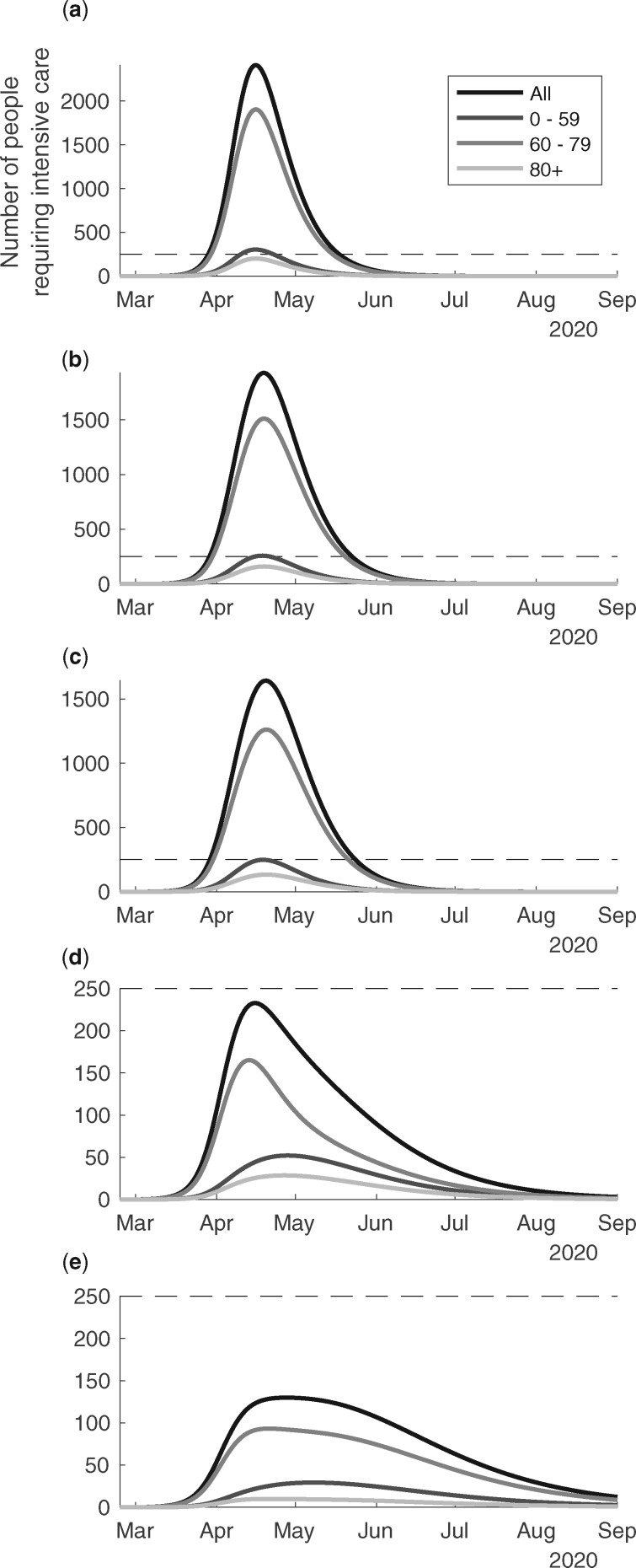
The predicted ICU bed demand per day from 24th of February to the 1st of September, 2020, in the region of Stockholm in relation to different suppression & mitigation scenarios. The scenarios are organized in rows with panel (**a**) no public health interventions (counterfactual scenario); (**b**) modest physical distancing in ages 0–59 years, moderate in ages 60+ years; (**c**) modest physical distancing in ages 0–59 years, moderately strong in ages 60+ years; (**d**) moderate physical distancing in ages 0–59 years, very strong in ages 60–79 years, strong in ages 80+ years, and with an increased degree of isolation of infectious individuals; (**e**) moderate physical distancing in ages 0–59 years, strong in ages 60+ years, and further improved isolation of infectious individuals. Mitigation giving rise to these predicted values had onset the 20th of March.

In Figure 3 we present the scenarios of country level COVID-19 ICU bed demand over time in Sweden for the different age groups, and in total. According to scenario (a), the outbreak would peak at the end of April and reach an ICU bed demand >10 000 patients (Figure 3a). The group <60 years of age alone would take up more than the baseline ICU resources of 526 beds during a month at the peak. According to scenario (b), the ICU demand would peak at around 9000 beds at the peak around 1 May (Figure 3b). The demand would be flattened and continue for a longer period. The ICU demand for those <60 years of age would again exceed the baseline ICU beds for 1 month. According to scenario (c), the ICU bed demand would peak at ∼7000 at the peak around 1 May (Figure 3c). The demand would be flattened and continue for a longer period. The ICU bed demand for those aged <60 years would almost take up the baseline ICU beds for a period slightly <1 month. According to scenario (d), the intensive care demand would increase just beyond 500, it would peak in April and decrease gradually to low levels in August (Figure 3d). According to scenario (e), the intensive care demand would be <300 for the whole study period (Figure 3e). We note that the outbreak would likely resurge unless there were other means were to control the transmission when the countermeasures are lifted in scenarios (d) and (e). Corresponding estimates for the Stockholm region are given in Figure 4a–e. It is noteworthy that the healthcare demand peaks slightly earlier in Stockholm as compared with Sweden as a whole.

In Supplementary Information 2, available as Supplementary data at *IJE* online, we show how the timing of the increase in cases across the scenarios (a)–(e) presented in Figure 2 is sensitive to mobility between municipalities (Supplementary Figure S2.1, available as Supplementary data at *IJE* online). There is a slightly earlier increase in ICU bed demand with higher inter-municipality mobility.

In Table 2 we describe the predictions of the total number of individuals infected, the total person days of care, the total person days of ICU occupancy, the total number of deaths (assuming all ICU demands are satisfied) and the deaths from ICU capacity shortage (100% above baseline level). Following on this, we estimate the direct costs of the care and intensive care demands (Table 3). The number of infected individuals in Sweden is predicted very high by the model in scenarios (a)–(c) with the cumulative number of infected people in the population already >90% by 1 September 2020 (Table 2). In scenarios (d) and (e), only 2.4 and 1.7 million people, respectively, would be infected by 1 September.

**Table 2:** Estimates of infections and healthcare demand aggregated over Sweden for the period 24 February to 1 September 2020, assuming intensive care capacity in Sweden increases by 100%, i.e. 1052 ICU beds in Sweden available for COVID-19 patients

Mitigation and suppression actions	Total number of individuals infected	Person days in in-patient care	Person days in the ICU	Total number of deaths assuming all ICU demands are satisfied	Total number of deaths from ICU shortage
(a) No public health interventions (counterfactual scenario)	10 093 341	993 119	347 315	41 104	28 906
(b) Modest physical distancing in ages 0–59 years, moderate in ages 60+ years	9 661 307	938 429	324 881	43 213	25 424
(c) Modest physical distancing in ages 0–59 years, moderately strong in ages 60+ years	9 273 896	866 471	290 496	38 889	21 451
(d) Moderate physical distancing in ages 0–59 years, very strong in ages 60–79 years, and strong in ages 80+ years, and improved isolation of infectious individuals	2 477 887	171 266	41 231	8477	0
(e) Moderate physical distancing in ages 0–59 years, strong in ages 60+ years, and further improved isolation of infectious individuals	1 706 342	126 854	34 445	4912	0

**Table 3:** Estimates of direct costs of infections and healthcare demand aggregated over Sweden for the period 24 February to 1 September 2020

Mitigation and suppression actions	Costs of cumulative person days in in-patient care (million SEK/2020)	Costs of cumulative person days in intensive care (million SEK/2020)	Total direct healthcare costs (million SEK/2020)
(a) No public health interventions (counterfactual scenario)	15 000	11 000	26 000
(b) Modest physical distancing in ages 0–59 years, moderate in ages 60+ years	15 000	10 000	25 000
(c) Modest physical distancing in ages 0–59 years, moderately strong in ages 60+ years	13 000	9000	22 000
(d) Moderate physical distancing in ages 0–59 years, strong in ages 60+ years, and improved isolation of infectious individuals	3000	1000	4000
(e) Moderate physical distancing in ages 0–59 years, strong in ages 60+ years, and further improved isolation of infectious individuals	2000	1000	3000

Overall, the scenarios show that the demand on inpatient care varies from just <1 000 000 person-days to just >100 000 person-days, whereas the demand on intensive care ranges from ∼350 000 to ∼30 000 person-days. The death rates, assuming no limits in ICU, varies from ∼40 000 to 5000 depending on the mitigation and suppression actions. Assuming instead a cap on the ICU bed capacity of 100% above baseline, the estimated number of additional excess deaths from lack of ICU capacity varies from 29 000 to 0 depending on the scenario. The total direct medical cost ranges between 26 billion and 3 billion Swedish krona (SEK) depending on the scenario (Table 3).

A statistical time series analysis of the total all-cause mortality in Stockholm indicates that due to possible misdiagnosis, an additional 0.40 (95% CI: 0.24, 0.57) deaths could be attributed to covid-19 and added to the confirmed COVID-19 cases (Supplementary Information 3).

## Discussion

Our study shows an exponential growth of the number of COVID-19 infections, health care demands and deaths in Sweden which became apparent towards the end of March and the beginning of April 2020. In April, it further suggests a strong effect of the physical distancing efforts put into place successively from around the middle of March in Sweden. The epidemiological data from Sweden currently align best to our modelled secenario (d) which describe a moderate physical distancing in those <60 years of age, a very strong distancing of those between 60 and79 years of age, strong distancing for ages 80+ years, and improved awareness and compliance of home isolation of symptomatic COVID-19 cases. So far, the level of physical distancing and isolation has not seriously compromised access to health care and has not overwhelmed the health care system. The policy and measures were less stringent and economically damaging compared with those introduced in other Scandinavian countries (Norway, Finland, Denmark), but the number of deaths per capita by early June 2020 was much higher. According to the European Centre for Disease Control (ECDC) situation update worldwide published by the ECDC on 8 June 2020, there were 461 deaths with 4429 confirmed infections per million inhabitants in Sweden, versus 102 deaths with 2063 reported cases in Denmark, 44 deaths with 1569 cases in Norway and 58 deaths with 1260 cases in Finland per million inhabitants.^19^ In the counterfactual scenario (e.g. no public health interventions), the intensive care unit demand was estimated to be >20 times higher than the intensive care capacity in Sweden and the number of deaths would be between 40 000 and 70 000.

If the policy and behaviour change would continue according to the development in scenario (d), our estimates using the Swedish model of physical distancing without lockdown show that by the end of August, 2020 just <25% of the Swedish population will have been infected, resulting in just >8000 deaths by that time point. Despite such high exposure, Sweden would remain below the herd immunity projected to stop the outbreak, a threshold estimated to be in the range 40–70% assuming no pre-existing immunity.^20^^,^^21^ Therefore, up to September 2020, the predicted impact is very dependent on ongoing adherence to physical distancing and shielding of vulnerable age groups. If such measures are not maintained, the consequences would be severe with demands substantially exceeding health care capacity and mortality rates rapidly increasing. Natural herd immunity, i.e. a situation where $R_{0}$ goes below 1 even if countermeasures were discontinued, appears not to be a viable objective to stop the virus circulation given the predicted death tolls. A potential glimmer of light, in such a scenario, is if some individuals previously not exposed to SARS-CoV-2 can express T-cell reactivity, contributing to some immunity.^22^ Further measures, including enhanced testing, prompt isolation of cases, more effective contact tracing and quarantining of contacts,^23^^,^^24^ would result in further reducing transmission intensity and daily new cases, while avoiding lockdown, until other control options such as vaccine and effective therapeutic options are readily available. We note, that scenario (d) should not be seen as a forecast of the development of the epidemic in Sweden, as it assumes that policy and behaviour (physical distancing, mobility and home isolation) would remain the same as during the last 2 months of the study period, which is unrealistic.

Of note, due to the strong triage in our model with only 15% of those aged 80+ being treated at the ICU, the demand of this group is overall already estimated to be very low. In order to capture the Swedish mortality patterns, our model further estimates a substantial number of deaths occurring outside hospitals, mainly in care homes. The excess mortality estimates published by the ECDC indicate a substantial amount of additional deaths must have occurred in Europe due to COVID-19.^25^ The total all-cause mortality in Stockholm indicate that a confirmed COVID-19 death is associated with an additional 0.40 (95% CI: 0.24–0.57) all-causes death, e.g. 40% additional deaths beyond the confirmed COVID-19 cases reported.

In Sweden, we calibrated the rates of in-patient care and critical care to be lower than those by Ferguson *et al*.^13^ for the UK (See Supplementary Table S1.2). Our results show that the demand on ICU beds can be reduced not only by a suppression strategy as successfully used in China, but also by a mitigation strategy. However, deaths cannot be effectively prevented in mitigation scenarios as many of those would occur independent of ICU demands. We note that, for the modes of death possible in our model (see Supplementary Table S1.3, available as Supplementary data at *IJE* online), the ICU demand can be maintained at a lower level while deaths rise, which appears to align well with the Swedish reported data including a substantial number of deaths occurring outside hospitals.

Our analyses address the impacts from COVID-19 on the health-care demand, deaths and direct healthcare costs in Sweden in relation to different public health interventions. As such it is in line with the assessment of the European Centre for Disease Prevention and Control regarding the COVID-19 pandemic. We find that the direct health-care related costs are substantial, ranging between 3 and 26 billion SEK dependent on the scenario. We show that more stringent mitigation or suppression efforts yield larger direct health care cost reductions compared with less stringent mitigation or suppression, or the counterfactual scenario, with the maximum cost difference estimated to be 23 billion SEK. These cost estimates are likely an underestimation of the total costs because they only estimate the direct costs within health care. Further on, the costs of the health sector would need to be balanced against the cost to the economy as a whole. The estimates here do not capture impacts within healthcare from other acute health-problems for which treatment is down prioritized or postponed due to the acute situation of the epidemic. Furthermore, they does not consider economic impacts beyond the health sector.

In the model we do allow *R_e_* to vary between municipalities and over time in response to the scenarios. However, we do not allow substantial inhomogenity in *R_e_*, so called superspreading, which could perhaps explain some of the clustering patterns and events associated with the COVID-19 pandemic.^27^ This is a limitation of our study, and if superspreading is really an important characteristic of COVID-19, our study does not capture such dynamics or its response to the countermeasures in the scenarios. However, with many people infected in a population, the role of superspreading becomes less important as opportunities for spread cease.^28^ We also did not consider the role of seasonality, and it may be that we are failing to predict a slowing down of transmission in the summer and a corresponding risk of resurgence during the winter season. Further on, underreporting, or asymptomatic transmission, can be a driver of herd-immunity. Preliminary findings from Iceland found no more than 50% asymptomatic carriers.^29^ Ferguson *et al*. estimated an Case Fatality Ratio (CFR) of ∼1.6% and and IFR of ∼0.8% based on a 50% asymptomatic rate.^13^ A Swedish study (the same as the PCR based study used for the validation of the model), prospectively measured and observed an IFR of ∼0.6%. The study was based on a random sample from the Stockholm region where individuals that were PCR positive for SARS-CoV-2 were followed up and the death frequency recorded.^30^ Interestingly, our study estimates and IFR are in agreement with this estimate when accounting for the excess mortality of ∼40%.

Our study supports that public health interventions such as social distancing combined with shielding of older persons, even without a strict lockdown, can protect the health care system by not exceeding ICU capacities. This intervention strategy, however, thus far resulted in higher disease burdens and deaths as compared with neighbouring countries with similar population densities that introduced more stringent lockdown measures. In the longer run, it will be important to evaluate both the health, society and economic impacts of contrasting mitigation and suppression policies to identify the best response to future pandemics.

## Supplementary data

Supplementary data are available at *IJE* online.

## Conflict of Interest

None declared.

## Supplementary Material

dyaa121_Supplementary_Data
