# Erratum to: COVID-19 healthcare demand and mortality in Sweden in response to non-pharmaceutical mitigation and suppression scenarios

**DOI:** 10.1093/ije/dyaa234

**Published:** 2020-11-22

**Authors:** Henrik Sjödin, Anders F Johansson, Åke Brännström, Zia Farooq, Hedi Katre Kriit, Annelies Wilder-Smith, Christofer Åström, Johan Thunberg, Mårten Söderquist, Joacim Rocklöv

First published online: 20 September 2020, *Int J Epidemiol* 2020; doi: https://doi.org/10.1093/ije/dyaa121

Upon the original publication of this article, the publication history was incorrect.  This has been corrected to read: “Received 29 March 2020; Editorial decision 18 June 2020; Accepted 19 June 2020”. The publisher apologises for the error.

