# Supplementary material for: COVID-19 healthcare demand and mortality in Sweden in response to non-pharmaceutical mitigation and suppression scenarios
Source: Int J Epidemiol. 2020 Sep 20:dyaa121. doi: 10.1093/ije/dyaa121 (PMC7543571; doi:10.1093/ije/dyaa121)
Supplement: dyaa121_Supplementary_Data [file dyaa121_supplementary_data.docx]

**Supplementary Information**

**Supplementary Information 1: Compartmental model and parameterization**

To account for time delays in the spatial spread of SARS-CoV-2 over the large geographical ranges in Sweden, we set up a spatial compartmental model. We distinguish between local (municipality) and global (Sweden) processes. The effects of local contact structures are assumed to be well described by the law of mass action; at local scales we assume a well-mixed contact-structure. The effects of global contact structures are assumed to be well described by a radiation model,(1) which gives rise to time delays in the spatial progression of infections over Sweden.

*Extended SEIR-model*

We apply an age-structured SEIR-based compartmental model for each municipality. In each municipality $i$, we account for all individuals that are susceptible $S_{i}$; latent (exposed) $E_{i}$; infectious but not going into healthcare $I_{i}$; infectious and going into some form of hospital care $J_{i}$; in healthcare $H_{i}$; in intensive care $C_{i}$; recovering in healthcare after critical care $\tilde{H}_{i}$; dead due to SARS-CoV-2 infection $D_{i}$; still infected but not transmitting to others e.g., isolated or otherwise removed so to not potentially transmit $\tilde{R}_{i}$, or, recovered $R_{i}$. Each respective variable is age-structured (i.e., vectors with age-specific component values). We account for three age-classes, $a=\left( 0-59, 60-79, 80+ \right)$ years. The compartmental model, with dot-notation for time derivatives, can then be written

| $\dot{S}_{i}=-\beta\left( \frac{S_{i}\left( 1-\sum_{j\neq i} t_{ji} \right)\left( \left( \sum_{a} \left( I_{i}+J_{i} \right) \right)+\sum_{i\neq j} \left\langle T_{I} \right\rangle_{ij}-\sum_{j\neq i} \left\langle T_{I} \right\rangle_{ji} \right)}{N_{i}}+S_{i}\sum_{j\neq i} \frac{t_{ji}\left( I_{j}+J_{j} \right)}{N_{j}} \right)$ $\dot{E}_{i}=\beta\left( \frac{S_{i}\left( 1-\sum_{j\neq i} t_{ji} \right)\left( \left( \sum_{a} \left( I_{i}+J_{i} \right) \right)+\sum_{i\neq j} \left\langle T_{I} \right\rangle_{ij}-\sum_{j\neq i} \left\langle T_{I} \right\rangle_{ji} \right)}{N_{i}}+S_{i}\sum_{j\neq i} \frac{t_{ji}\left( I_{j}+J_{j} \right)}{N_{j}} \right)-\frac{E_{i}}{p_{E}}$ $\dot{I}_{i}=\frac{\left( 1-\epsilon_{i} \right)E_{i}}{p_{E}}-\frac{I_{i}}{p_{I}}$ $\dot{J}_{i}=\frac{\epsilon_{i}E_{i}}{p_{E}}-\frac{J_{i}}{p_{J}}$ $\dot{H}_{i}=\frac{\left( 1-\chi_{i} \right)J_{i}}{p_{J}}-\frac{H_{i}}{p_{H}}$ $\dot{C}_{i}=\frac{{\left( 1-\tau\right)\chi}_{i}J_{i}}{p_{J}}-\frac{C_{i}}{p_{C}}$ ${\dot{\tilde{H}}}_{i}=\frac{\left( 1-\mu\right)C_{i}}{p_{C}}-\frac{\tilde{H}_{i}}{p_{\tilde{H}}}$ $\dot{D}_{i}=\frac{\mu C_{i}}{p_{C}}+\frac{\tau\chi_{i}J_{i}}{p_{J}}+\frac{\nu I_{i}}{p_{I}}$ $\dot{\tilde{R}}=\frac{{\left( 1-\nu\right)I}_{i}}{p_{I}}-\frac{\tilde{R}}{p_{\tilde{R}}}$ $\dot{R}_{i}=\frac{\tilde{R}}{p_{\tilde{R}}}+\frac{H_{i}}{p_{H}}+\frac{\tilde{H}_{i}}{p_{\tilde{H}}}$ | (S1.1) |
| --- | --- |

where $N_{i}=S_{i}+E_{i}+I_{i}+J_{i}+\tilde{R}_{i}+R_{i}$, and see table S1.1 for model parameterization. Infection are carried between municipalities, and $\left\langle T_{I} \right\rangle_{ij}$ denotes the number per day of infected individuals that are resident to the $j$th municipality and are visiting the $i$th municipality; $S_{i}\sum_{j\neq i} t_{ji}$ denotes the number per day of susceptible individuals that are resident to the $i$th municipality and are visiting other municipalities. This should be seen as daily averages.

*Radiation model*

These mobility rates are given by a radiation model^1^, where we used the time dependent rate scaling $\alpha(t)$, with 0.01 as the baseline, i.e., the counter-scenario of inter-municipality travel-reductions. The radiation model for the average number (denoted by angle-brackets) of travellers per day of $X_{i}$, from municipality $i$ to municipality $j$, can be written $\left\langle T_{X} \right\rangle_{ji}={X_{i}t}_{ji}$, with the per person travel-probability

$$t_{ji}=\alpha\left( t \right)\frac{n_{i}n_{j}}{\left( n_{i}+s_{ji} \right)\left( n_{i}+n_{j}+s_{ji} \right)},$$

where $n_{i}$ is the number of citizens in the municipality $i$; where $s_{ji}$ is the total population size within a circle with a radius equal to the distance between two municipalities $i$ and $j$. Note that $t$ without subscript denotes time. See table S1.1 for $\alpha(t)$.

*Geographic and demographic data*

Population data were collected from the Statistics Sweden and the demographical geographical statistical units’ database. The database provides population data in 5-year age categories for almost 6000 administrative areas in Sweden and was compiled by the end of 2018. We aggregated this population data to the municipal level and into three age-groups: 0-59, 60-79 and older than 79 (i.e., 80+) years.

The geographical centroid coordinates (latitude, longitude) of municipalities were derived from shape-file data by using the R software libraries sp, rgdal, rgeos and foreign. These coordinates were used to calcluate the distances between each and all of the 290 Swedish municipalities. A distance matrix was constructed, and used to derive inter-municipality travel rates given by the radiation model.

*Heterogeneous basic reproduction number (*$R_{0})$

For the SEIR-model formality without vital dynamics, $R_{0}$ is equal to $\beta$ times the infectious period. As our model extends in some aspects from the stylized SEIR-model, $R_{0}$ in our model is approximately given by this product. $R_{0}$ depends on the contact-rate and infectious period. As both of these parameters are age-dependent, and that each municipality has unique age-distributions, our model accounts for a heterogeneous $R_{0}$ which vary between municipalities. The age-dependence for contact rates comes from our assumptions on th within-municipality contact-structure (Supplementary Information 2), and relate directly to our modelled and presented scenarios. The age-dependence for infectious period (i.e., in this context, the number of days an infected individual can infect other individuals), arises here indirectly from that the length of the infectious period differs between those individuals that go into healthcare (and being practically isolated) and those that do not go into heathcare, and the proportion among infected individuals in ether of these two groups is age-dependent. Accordingly, $R_{0}$ per municipality , without counter measures (i.e., for scenario a), varies within the interval 2.67 and 4.45. The low extreme would imply a three-days infectious period if all infected individuals go into healthcare, and the upper extreme would imply a five-days infectious period if no infected individuals go into health care. As the proportion of infected individuals going into healthcare is never zero nor one (Table S1.2), we know that none of the extremes in the interval becomes realized, yet that $R_{0}$ is within the interval $[2.67, 4.45]$.

*Numerical analysis and calibration procedure*

The equation system (eq. S1.1) was solved in Matlab for respective variables by implementing the ordinary differential-equation solver ode45, with the initial condition that all individuals were susceptible except for 1/50000 of the population in Region Stockholm and 1/100000 of the population in all other Swedish Health-care regions (and municipalities), respectively.

The solved variables could accordingly be compared against empirical data on deaths, ICU load, healthcare load, virus-prevalence data and seroprevalunce. The model parameterization was iteratively calibrated agaist these data with the objective to narrow down parameter-values to fit the dynamics of one of the five scenarios to these data (for R^2^- and mse-values, see Table 1 in the main text). Data on COVID-19 deaths- and hospitalization were obtained through various online data aggregating services, (3,4) Data regarding healthcare load, ICU occupancy, and confirmed deaths were reported directly from the healthcare regions as well as the Swedish Public-Health Agency. The virus carriage data were fetched from a study performed by the Swedish Public-Health Agency.(5) The seroprevalence data reporting 7.3% in early April was also from the Swedish Public-Health Agency.

*Deriving Swedish COVID-19 hospitalization and death frequencies*

Drawing on age-structured data^2^ on the proportion of reported cases requiring inpatient care (TableS1.2), we derived the corresponding *proportion of infected cases requiring hospitalization* ($\epsilon_{i}$) for any municipality $i$ and for age-classes $a=(0-59, 60-79, 80+)$ by taking a weighted average for each age-class in $a$ and multiplying by 1/6 (obtained by calibration to Swedish data) We also derived the *proportion of hospitalized cases requiring intensive care* by the weighted averaging. Note that we further assumed a 85% and a 10% intensive-care triage for the ages 60-79 and 80+ years, respectively.

Mortality risk among the group of individuals receiving intensive care (i.e., $\mu_{i}$; Table S1.1) was derived by forming weighted averages of mortality risk for age-groups 0-59, 60-79 and 80+, based on the observed mortality risk in Italy.(6) We assume that the mortality risk in inpatient care outside the ICU is zero (a critically ill patient would be transferred to the ICU), and that the mortality risk among the group of individuals that is not admitted to the healthcare system (for different reasons), is proportional by a factor 0.0069 (estimated by calibration to Swedish data) to the ICU mortality-risk distribution. We assume that patients that are not prioritized for care in the ICU based on the medical ethical principles set up in Sweden the pandemic will soon die from COVID-19.(7) The resulting country-level mortality-risk in Sweden for infected individuals in age-groups 0-59, 60-79 and 80+ is presented in Table S1.3.

*Scenarios and within-municipality contact structures*

Table S1.4 provides overall contact-rate scaling $\hat{c}$ within and between age groups. They can be seen as reduction coefficients to $R_{0}$ as a result of physical distancing. They can be interpreted as probabilities of exposure to potential contacts (e.g., the probability of going to public places, or equivalent, and not keeping safe distancing) times the contact-rate $q$ given exposure to potential contacts (i.e., the contact-rate at public places), where the latter was normalized so that the overall contact-rate scaling equals to 1 without any suppression measures. Specifically, $\hat{c}$ is equal to $p_{a}p_{b}q$ for any age groups $a$ and $b$, where $p_{a}$and $p_{b}$ are probabilities that any individual from age group $a$ and $b$, respectively, expose themselves to situations where potential contacts are possible. This can be generally expressed by a symmetrical “contact-matrix”

$$q\left( \begin{matrix} p_{1}^{2} & p_{1}p_{2} & p_{1}p_{3} \\ p_{1}p_{2} & p_{3}^{2} & p_{2}p_{3} \\ p_{1}p_{3} & p_{2}p_{3} & p_{3}^{2} \end{matrix} \right),$$

which is provided in Table S1.4 paremerizised for each respective scenarios a to e (e.g., figure 3 in main text), where any entries left of the main diagonal was omitted due to symmetry. Table S1.4 presents exactly the modelled scenarios a to e, with the only exception that scenario d and e in addition account for increased isolation of infectious individuals; modelled by reductions, 29% and 40%, respectively, in the infectious period among individuals not going into healthcare. The different scenarios are generally described by contact rates according to:

- a) no changes in contact rates (baseline model);
- b) 25% reduction in contacts in ages 0-59 years and 50% reduction contact in ages 60+ years.
- c) 25% reduction in contacts in ages 0-59 years and 75% reduction in ages 60+ years;
- d) 56% reduction in contacts in ages 0-59 years; 98% reduction in contacts in ages 60-79 years and 72% reduction in ages 80+ years, and additionally, a reduction of infectious period from 5 days to to 3.5 days among the general infected population by home isolation;
- e) 50% reduction in contacts in ages 0-59 years and 90% reduction in ages 60+ years and further reduction of infectious period to 3.0 days among the general infected population by home isolation.

See Table S1.4 for the between age group contact rate reductions for scenario (a)-(e).

| **Table S1.1.** Parameters in equation system S1.1 and their respective values. | | | |
| --- | --- | --- | --- |
| Parameter | Notation and value | Unit | Notes |
| Age-classes | $a=\left( 0-59, 60-79, 80+ \right)$ | Years |  |
| Transmission rate | $\beta=0.89 c\left( t \right)$ | Days^-1^ | Calibration to Swedish data. |
| Latent period | $p_{E}=4$ | Days |  |
| Infectious period | $p_{I}=5$ | Days | $p_{I}=3.5$ and $p_{I}=3$ in scn. d and e. Scn. d from calibration to Swedish data. |
| Pre-hospitalization infectious period | $p_{J}=3$ | Days |  |
| Hospitalization period | $p_{H}=7$ | Days |  |
| Intensive care period | $p_{C}=10$ | Days |  |
| Post intensive -care period | $p_{\tilde{H}}=7$ | Days |  |
| Removed period | $p_{\tilde{R}}=12-p_{I}$ | Days |  |
| Proportion of infected cases requiring hospitalization | $\epsilon_{i}$ |  | See table S1.2 for age structured values. Calibration to Swedish data. |
| Proportion of hospitalized cases requiring intensive care | $\chi_{i}$ |  | See table S1.2 for age structured values. Calibration to Swedish data. |
| Proportion of respective age-group in intensive care that dies | $\mu=\left( 0.13, 0.33, 0.55 \right)$ |  | Weighted averages based on Table 2 in (6) |
| Proportion of non-hospitalized individuals that dies. | $\nu=0.0069 \mu$ |  | Calibration to Swedish data. |
| Triage proportion | $\tau=(0, 0.1, 0.85)$ |  |  |
| Contact rate | $c\left( t \right)=c_{0}\left( \frac{1-\hat{c}}{1+\exp\left( \frac{5}{18}\left( t-\hat{t} \right) \right)}+\hat{c} \right)$ | Days^-1^ | Reduced to $\hat{c}$ around time $\hat{t}$ with $c_{0}=\left( 1, 1, 1 \right)$for age-groups. Calibration to Swedish data |
| Inter-municipality travel-rate scaling | $\alpha\left( t \right)=\alpha_{0}\left( \frac{1-\hat{\alpha}}{1+\exp\left( \frac{5}{18}\left( t-\hat{t} \right) \right)}+\hat{\alpha} \right)$ | Days^-1^ | Reduced to $\alpha_{0}\hat{\alpha}$ around day $\hat{t}$; with $\alpha_{0}=0.01$. Calibration to Swedish data |
| Onset time of intervention | $\hat{t}=\mathrm{March} 29th, 2020$ | Days | Mean intervention realization of countermeasures initiated around March 20th, 2020. Calibration to Swedish data |
| Contact rate scaling | $\hat{c}$ | Days^-1^ |  |
| Travel rate scaling | $\hat{\alpha}=0.1$ | Days^-1^ | $\hat{\alpha}=1$ in figure S2.1. |

| **Table S1.2.** Conditional risks for in-patient care and intensive care | | | | |
| --- | --- | --- | --- | --- |
| Age-class (years) | From Ferguson^2^ | | Calibrated to Sweden & Stockholm | |
| Age-class (years) | Percent of reported cases requiring inpatient care | Percent of in-patient cases requiring intensive care | Percent of infected persons going into inpatient care | Percent of in-patient cases requiring intensive care |
| 0-9 | 0.1 | 5.0 | 0.02 | 5.0 |
| 10-19 | 0.3 | 5.0 | 0.06 | 5.0 |
| 20-29 | 1.2 | 5.0 | 0.26 | 5.0 |
| 30-39 | 3.2 | 5.0 | 0.69 | 5.0 |
| 40-49 | 4.9 | 6.3 | 1.06 | 6.3 |
| 50-59 | 10.2 | 12.2 | 2.20 | 12.2 |
| 60-69 | 16.6 | 27.4 | 3.59 | 27.4 |
| 70-79 | 24.3 | 43.2 | 5.25 | 43.2 |
| 80+ | 27.3 | 70.9 | 5.90 | 70.9 |

| **Table S1.3.** Mortality risk among infected populations (%) | | | |
| --- | --- | --- | --- |
| Age-class (years) | In intensive care | After triage prior to intensive care | Outside of hospital-care |
| 0-59 | 0.0067 | 0 | 0.09 |
| 60-79 | 0.45 | 0.15 | 0.22 |
| 80+ | 0.34 | 3.55 | 0.35 |

| **Table S1.4**: Contact rate scaling, i.e., $\hat{c}$ (Table S1.1), by scenarios (a) to (e). Values in (d) from calibration to Swedish data. | | | | |
| --- | --- | --- | --- | --- |
| Scenario | Age Group | 0-59 | 60-79 | 80+ |
| a | 0-59 | 1 | 1 | 1 |
|  | 60-79 |  | 1 | 1 |
|  | 80+ |  |  | 1 |
| b | 0-59 | 0.75 | 0.61 | 0.61 |
|  | 60-79 |  | 0.50 | 0.50 |
|  | 80+ |  |  | 0.5 |
| c | 0-59 | 0.75 | 0.43 | 0.43 |
|  | 60-79 |  | 0.25 | 0.25 |
|  | 80+ |  |  | 0.25 |
| d | 0-59 | 0.44 | 0.083 | 0.35 |
|  | 60-79 |  | 0.015 | 0.066 |
|  | 80+ |  |  | 0.28 |
| e | 0-59 | 0.50 | 0.22 | 0.22 |
|  | 60-79 |  | 0.1 | 0.1 |
|  | 80+ |  |  | 0.1 |

**Supplementary Information 2: Scenario with no reduction in inter-municipality travel rates**

Figure S2.1 shows the results on ICU load should there be no reduction of inter-municipality travel rates, and with other things equal to the model parameterization for which Figure 3 in the main text is the result.

**Figure S2.1.** Scenarios of ICU demand simulated based upon higher mobility between municipalities as described by the radiation model.

**Supplementary Information 3: Analysis of excess mortality in Stockholm**

Using mortality data from Statistics Sweden (8) and officially confirmed COVID-19 mortality data reported by the 21 regions of Sweden collected up until the 11^th^ of April, 2020,(3) the excess mortality in response to reported COVID-19 deaths was estimated for the region of Stockholm using a linear regression model (see Figure S3.1). The outcome of this model was the daily total number of deaths in all-causes, subtracting the number of deaths observed in COVID-19. The number of deaths was assumed well described by a normal distribution due to the higher frequency of events. The analysis adjusted for time trends using a factor variable for year and season and a factor variable for month. The daily COVID-19 deaths were smoothed using a moving average with a window of 7 days. The excess mortality was modelled as a linear function and estimated of a linear increase of 0.40 (95% CI = 0.24, 0.57) increase in all-cause non-COVID-19 mortality for every COVID-19 death.

**Figure S3.1.** Deaths for weeks 8-10 and 13-15 in year 2018, 2019 and 2020 for the region of Stockholm, Sweden.

**Supplementary Information 4: Cost estimates**

The cost estimates were retrieved using Cost per Patient (CPP) database that reports costs for every individual care event. The database use Diagnose Related Group (DRG) to group the care events to be able to report costs that describe well the average costs at a group level. The database provides average time in care and the average costs per care event.(9)

*Average treatment costs calculation for inpatient care*

To account the costs per day in care, we used costs reported under following diagnose codes S.40 Virus infection; Main diagnoses: B.349 Virus infection, unspecified; J.108 Influenza due to other identified influenza virus with other manifestation. Average cost per day was calculated dividing the total cost with average number of days spent in care.

*Average treatment costs calculation for ICU care*

To calculate the ICU care all costs reported under respiratory diseases (D.20) receiving invasive ventilation treatment were extracted from the database and divided by average number of days for treatment to calculate the average cost per day.

For both inpatient and ICU care cost estimates, the total average cost for all age groups reported in 2018 was used. All cost estimates were adjusted for consumer price index and reported in 2020 SEK value. Cumulative patient days were multiplied with the average cost estimates for inpatient care and ICU care.

The Swedish Intensive Care Register Yearly Report 2018 estimates that an average cost per day in intensive care is from 50 000 – 80 000 SEK, which is a higher estimate that the one we are using here.(10) The lower estimate was chosen to avoid overestimations of direct costs, Notably, the COVID-19 costs for a proportion patients receiving extracorporeal membrane oxygenation treatment was not included here.

**References**

1. Simini, F., González, M.C., Maritan, A. & Barabási, A.-L. A universal model for mobility and migration patterns. *Nature 2020*, 484, 96–100.

2. Ferguson N, Laydon D, Nedjati Gilani G et al. Report 9: Impact of non-pharmaceutical interventions (NPIs) to reduce COVID19 mortality and healthcare demand. doi: https://doiorg/1025561/77482.

3. Epidemiological data: https://raw.githubusercontent.com/elinlutz/gatsby-map/master/src/data/time_series/time_series_deaths-deaths.csv, retrieved 2020-04-24

4. <https://www.dn.se/nyheter/grafik-det-nya-coronavirusets-utbredning-i-varlden/>, retrieved 2020-04-24

5.https://www.folkhalsomyndigheten.se/nyheter-och-press/nyhetsarkiv/2020/april/resultat-fran-undersokning-av-forekomsten-av-COVID-19-i-region-stockholm/, retrieved 2020-04-24

6. Grasselli, G., Pesenti, A. & Cecconi, M. (2020). Critical Care Utilization for the COVID-19 Outbreak in Lombardy, Italy: Early Experience and Forecast During an Emergency Response. *JAMA 2020*; 28;323(16):1545-6.

7. Swedish National Board for Health and Welfare: <https://www.socialstyrelsen.se/globalassets/sharepoint-dokument/dokument-webb/ovrigt/nationella-prioriteringar-intensivvarden.pdf>, retrieved 2020-04-24

8. Statistics Sweden: https://www.scb.se/om-scb/nyheter-och-pressmeddelanden/stockholm-sticker-ut-i-statistik-over-dodsfall/, retrieved 2020-04-24

9. Swedish Patient register for treatment costs: <https://statva.skl.se/SKL_KPP_information.html>

10. Annual report 2018, Swedish Intensive Care Register: <https://www.icuregswe.org/en/>, retrieved 2020-04-24
